# Small-animal SPECT/CT imaging of cancer xenografts and pulmonary fibrosis using a ^99m^Tc-labeled integrin αvβ6-targeting cyclic peptide with improved *in vivo* stability

**DOI:** 10.1007/s41048-018-0071-1

**Published:** 2018-11-02

**Authors:** Hao Liu, Liquan Gao, Xinhe Yu, Lijun Zhong, Jiyun Shi, Bing Jia, Nan Li, Zhaofei Liu, Fan Wang

**Affiliations:** 1 Medical Isotopes Research Center and Department of Radiation Medicine, School of Basic Medical Sciences, Peking University Health Science Center, Beijing, 100191 China; 2 Medical and Healthy Analytical Center, Peking University, Beijing, 100191 China; 3 Key Laboratory of Protein and Peptide Pharmaceuticals, CAS Center for Excellence in Biomacromolecules, Institute of Biophysics, Chinese Academy of Sciences, Beijing, 100101 China; 4 Key Laboratory of Carcinogenesis and Translational Research (Ministry of Education/Beijing), Department of Nuclear Medicine, Peking University Cancer Hospital & Institute, Beijing, 100142 China

**Keywords:** Integrin αvβ6, Pancreatic cancer, Pulmonary fibrosis, Molecular imaging, Peptide cyclization, Single-photon emission computed tomography (SPECT)

## Abstract

**Abstract:**

Integrin αvβ6 is expressed at an undetectable level in normal tissues, but is remarkably upregulated during many pathological processes, especially in cancer and fibrosis. Noninvasive imaging of integrin αvβ6 expression using a radiotracer with favorable *in vivo* pharmacokinetics would facilitate disease diagnosis and therapy monitoring. Through disulfide-cyclized method, we synthesized in this study, a new integrin αvβ6-targeted cyclic peptide (denoted as cHK), and radiolabeled it with ^99m^Tc. The ability of the resulting radiotracer ^99m^Tc–HYNIC–cHK to detect integrin αvβ6 expression in pancreatic cancer xenografts and idiopathic pulmonary fibrosis was evaluated using small-animal single-photon emission computed tomography (SPECT)/computed tomography (CT). ^99m^Tc–HYNIC–cHK showed significantly improved *in vivo* metabolic stability compared to the linear peptide-based radiotracer ^99m^Tc–HYNIC–HK. ^99m^Tc–HYNIC–cHK exhibited similar biodistribution properties to ^99m^Tc–HYNIC–HK, but the tumor-to-muscle ratio was significantly increased (2.99 ± 0.87 vs. 1.82 ± 0.27, *P *< 0.05). High-contrast images of integrin αvβ6-positive tumors and bleomycin-induced fibrotic lungs were obtained by SPECT/CT imaging using ^99m^Tc–HYNIC–cHK. Overall, our studies demonstrate that ^99m^Tc–HYNIC–cHK is a promising SPECT radiotracer for the noninvasive imaging of integrin αvβ6 in living subjects.

**Graphical Abstract:**

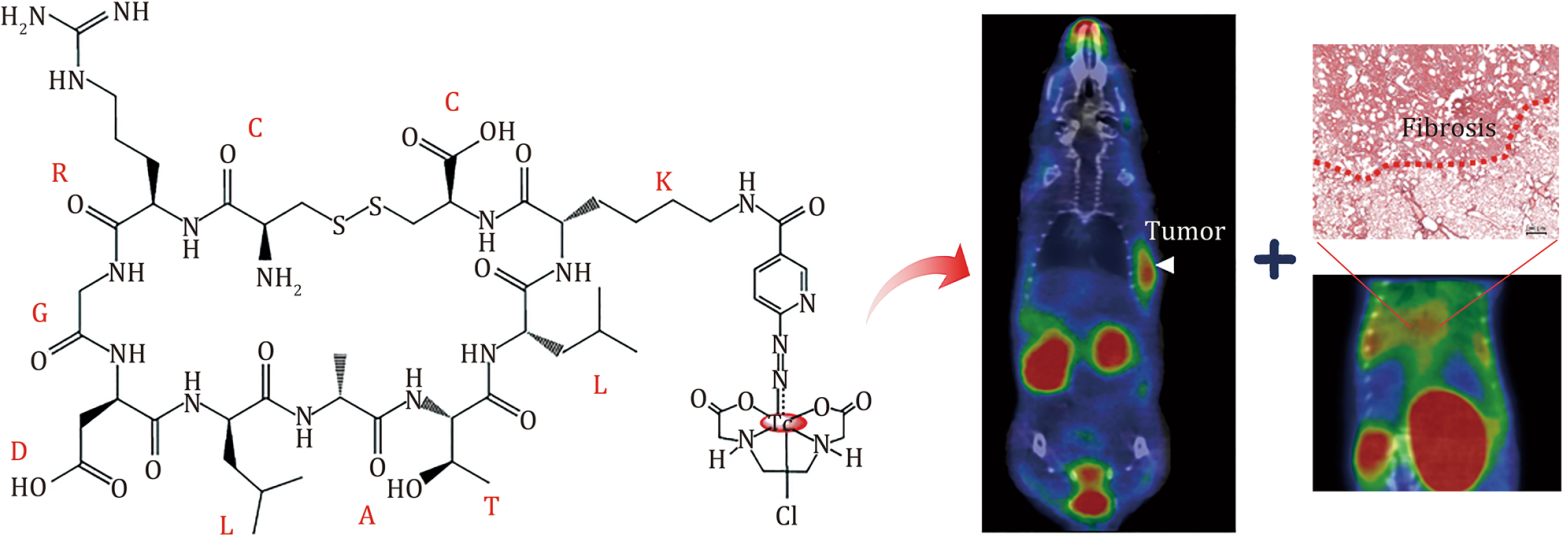

## Introduction

Integrin αvβ6, a member of the integrin family, is present at undetectable levels in adult differentiated tissues, but is overexpressed during embryogenesis, tumorigenesis, and tissue injury (Breuss *et al*. 1993; Desgrosellier and Cheresh 2010; Peng *et al*. 2016). Increased expression of integrin αvβ6 usually correlates with more aggressive disease and poor prognosis (Bates and Mercurio 2005; Elayadi *et al*. 2007; Lee *et al*. 2006), and the upregulation of integrin αvβ6 expression in a wide variety of cancers is associated with increased tumor cells migration, invasion, and metastasis (Bates 2005; Bates and Mercurio 2005). In addition to caner, de novo or increased expression of integrin αvβ6 has also been observed in pathological process of fibrosis (Patsenker *et al*. 2008; Pi *et al*. 2015). Idiopathic pulmonary fibrosis (IPF) is a chronic and progressive fibrotic lung disease with a poor prognosis (Gribbin *et al*. 2006; Navaratnam *et al*. 2011; Wells 2013). Integrin αvβ6-mediated transforming growth factor (TGF)-β activation has been implicated in multiple models of lung fibrosis, and the upregulated expression of integrin αvβ6 has also been found in patients with IPF (Horan *et al*. 2008; Xu *et al*. 2009). Importantly, the expression of integrin αvβ6 is temporally and spatially associated with the course of the fibrosis progression (Puthawala *et al*. 2008).

Due to the critical role of integrin αvβ6 in tumorigenesis and fibrogenesis, it has emerged as an appealing target for diagnostic imaging, prognosis evaluation, and therapeutic responses monitoring (Agarwal 2014; Cantor *et al*. 2015; Saini *et al*. 2015; Yang *et al*. 2015). Therefore, noninvasive and quantitative imaging of integrin αvβ6 expression by molecular imaging techniques would be of great potential for better management of these diseases. Previous studies have focused on the development of positron emission tomography (PET) and single-photon emission computed tomography (SPECT) radiotracers for *in vivo* imaging of integrin αvβ6 expression (Hackel *et al*. 2013; Hausner *et al*. 2007, 2008, 2009a, b, 2013; Hu *et al*. 2014; John *et al*. 2013; Kimura *et al*. 2012; Li *et al*. 2011; Liu *et al*. 2014b; Man *et al*. 2013; Nothelfer *et al*. 2009; Saha *et al*. 2010; Satpati *et al*. 2014; Singh *et al*. 2014; Ueda *et al*. 2014; Zhu *et al*. 2014). Although promising, most of these radiotracers are based on linear peptides, which have poor *in vivo* metabolic stability and suboptimal pharmacokinetics. For example, we observed recently that a ^99m^Tc-labeled linear peptide (RGDLATLRQLAQEDGVVGVRK, the HK peptide) completely degraded *in vivo* within 30 min after injection, leading to a very low tumor uptake and tumor-to-nontumor ratios (Liu *et al*. 2014b).

Peptide cyclization has been reported to be a powerful strategy to improve the stability of peptides by means of adopted resistance to enzymatic degradation (Besser *et al*. 2000; Bogdanowich-Knipp *et al*. 1999a, b; Gilon *et al*. 1991; Pakkala *et al*. 2007; Roxin and Zheng 2012; Shi *et al*. 2016). To overcome the limitations of ^99m^Tc-labeled HK peptide, in this study, we selected the first seven amino acid residues of the integrin αvβ6-targeting HK peptide and added a lysine residue at C-terminal in order to conjugate the chelator. We then cyclized it by adding a cysteine residue at N- and C-terminals, respectively, to generate a new peptide c (CRGDLATLKC, denoted as cHK). The peptide cHK was conjugated with the chelator sodium succinimidyl 6-(2-(2-sulfonatobenzaldehyde) hydrazono) nicotinate (HYNIC)-NHS and then radiolabeled with ^99m^Tc. The resulting radiotracer ^99m^Tc–HYNIC–cHK was evaluated *in vivo* as a SPECT radiotracer for imaging of integrin αvβ6 expression in both cancer and IPF mouse models.

## Results

### Chemistry and radiochemistry

**Fig. 1:**
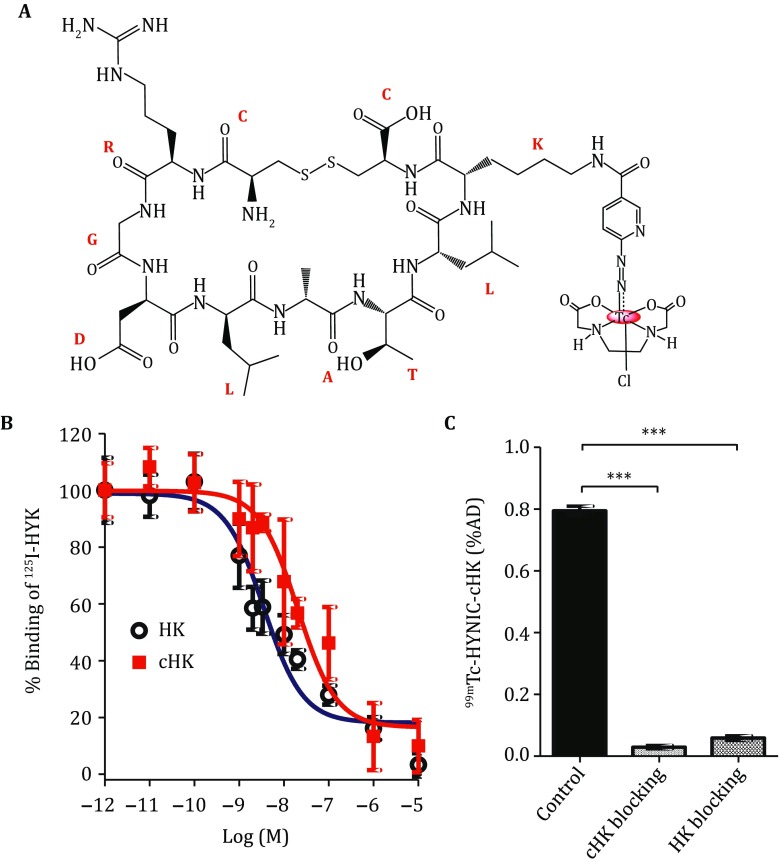
**A** Chemical structure of ^99m^Tc–HYNIC–cHK. **B** Inhibition of ^125^I–HYK binding to integrin αvβ6 on BxPC-3 cells by the cHK and HK peptides. **C** Binding of ^99m^Tc–HYNIC–cHK to BxPC-3 cells (without or with 300 μg of HK/cHK peptide blocking), ^***^P < 0.001

The Fmoc-cHK–HYNIC conjugate (Fig. 1A) was prepared by direct conjugation of Fmoc-cHK peptide with HYNIC-NHS. After the removal of Fmoc group, the final product HYNIC–cHK was confirmed by high-performance liquid chromatography (HPLC) and mass spectrometry. The HPLC purity of HYNIC–cHK was >95% before being used for ^99m^Tc radiolabeling. The ^99m^Tc-labeling procedure was done within 30 min with a yield ranging from 85%  to  90%. The radiochemical purity was >95% after purification, and the specific activity was >30 MBq/nmol.

### Cell-binding assay

Similar to the HK peptide, cHK peptide also inhibited the binding of ^125^I–RGDLATLRQLAQEDGVVGVRYK (HYK) to integrin αvβ6-expressing BxPC-3 cells in a concentration-dependent manner, but the integrin αvβ6 binding affinity of cHK was lower compared to that of HK peptide. The *IC*_50_ values for cHK and HK were 20.25 ± 1.17 and 3.55 ± 0.09 nmol/L, respectively (Fig. 1B).

The binding specificity of ^99m^Tc–HYNIC–cHK to integrin αvβ6 was evaluated in integrin αvβ6-positive BxPC-3 cells. As shown in Fig. 1C, the binding of ^99m^Tc–HYNIC–cHK to BxPC-3 cells was significantly inhibited by the addition of excess doses of the cHK and HK peptides (from 0.79 ± 0.01 %AD to 0.03 ± 0.005 and 0.06 ± 0.007 %AD, respectively, *P *< 0.001).

### Solution and metabolic stability

**Fig. 2:**
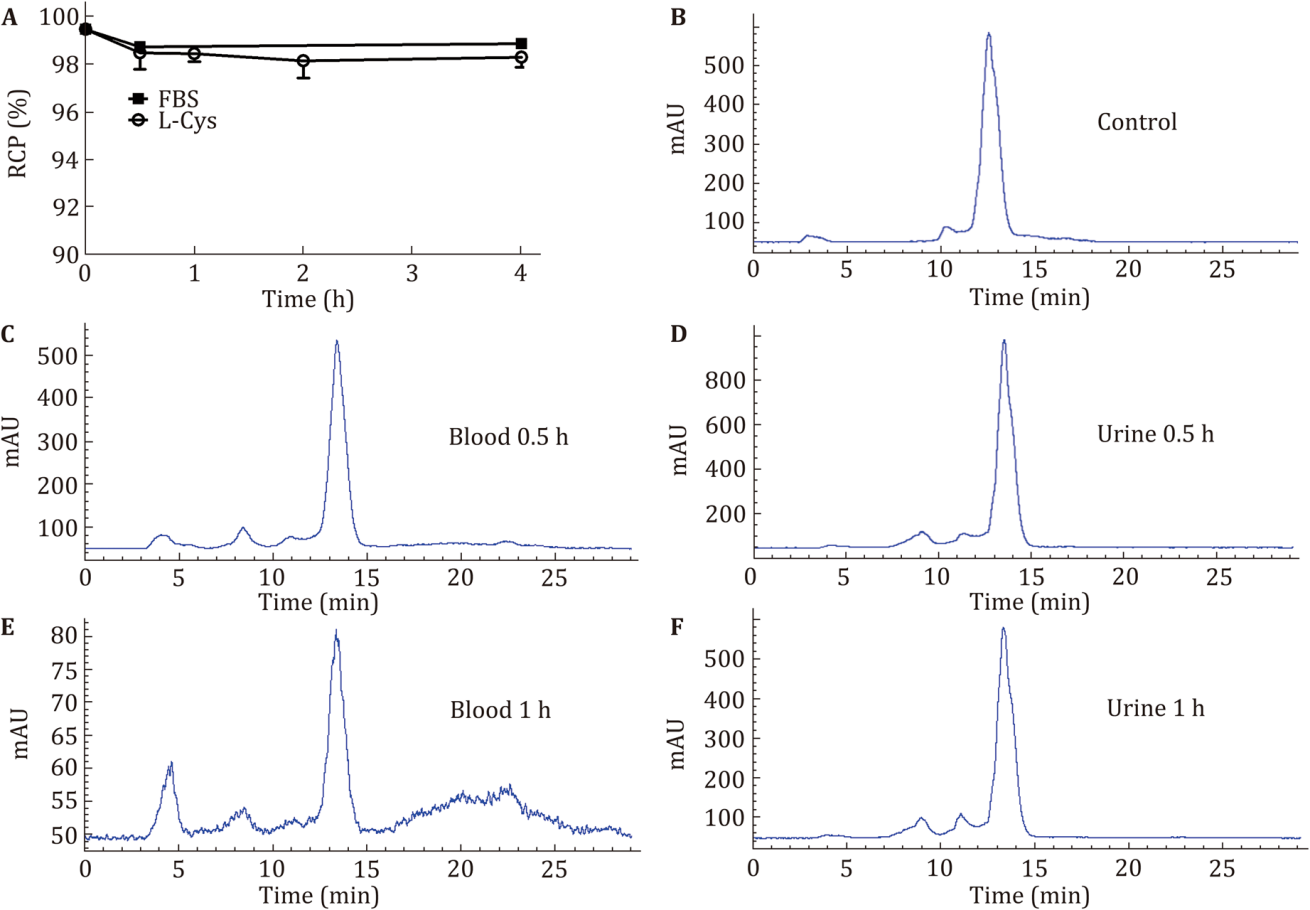
**A** Solution stability of ^99m^Tc–HYNIC–cHK in serum and l-cysteine (1.0 mg/mL). **B**–**F** Typical radio-HPLC chromatogram and metabolic stability of ^99m^Tc–HYNIC–cHK in mouse blood and urine at 0.5 and 1 h after injection

The *in vitro* solution stability of ^99m^Tc–HYNIC–cHK in fetal bovine serum (FBS) or l-cysteine was monitored by radio-HPLC. Figure 2A shows that ^99m^Tc–HYNIC–cHK remains stable for more than 4 h both in FBS and in the presence of l-cysteine.

We performed the metabolism studies of ^99m^Tc–HYNIC–cHK using normal BALB/c mice. We analyzed the samples from both blood and urine to determine whether the radiotracer retains its chemical integrity at 0.5 h and 1 h postinjection. Figures 2B–F illustrate the radio-HPLC chromatograms of ^99m^Tc–HYNIC–cHK before injection (Fig. 2B), in the blood (Fig. 2C, E) and in the urine (Fig. 2D, F). ^99m^Tc–HYNIC–cHK retained its integrity in urine, while showing the degrees of metabolism to be 9.94% and 30.09% in blood at 0.5 h and 1 h postinjection, respectively. Compared to the linear peptide-based radiotracer ^99m^Tc–HYNIC–HK (Liu *et al*. 2014b), ^99m^Tc–HYNIC–cHK is much more stable *in vivo*.

### Biodistribution

**Fig. 3:**
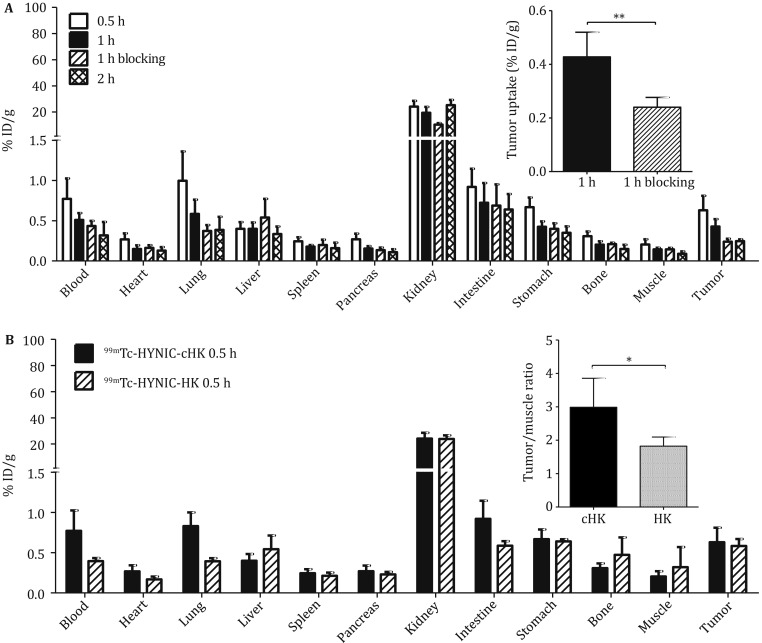
**A** Biodistribution of ^99m^Tc–HYNIC–cHK in BxPC-3 tumor-bearing nude mice at 0.5, 1, and 2 h after injection and coinjected with cold HK peptide at 1 h after injection. Inset: enlarged view of the tumor uptake values; ^**^P < 0.01. **B** Biodistribution of ^99m^Tc–HYNIC–cHK and ^99m^Tc–HHK in BxPC-3 tumor-bearing nude mice at 0.5 h after injection. Inset: enlarged view of the tumor-to-muscle ratios; ^*^P < 0.05

As shown in Fig. 3A, the uptake values of ^99m^Tc–HYNIC–cHK in BxPC-3 tumors were 0.63 ± 0.18, 0.43 ± 0.09, and 0.33 ± 0.16 %ID/g at 0.5, 1, and 2 h after injection, respectively. The tumor uptake of ^99m^Tc–HYNIC–cHK was higher than that in most of the normal organs, such as heart, liver, pancreas, bone, and muscle, at almost all time points examined (*P* < 0.05). The tumor uptake of ^99m^Tc–HYNIC–cHK was significantly reduced with a coinjection of an excess dose of the cold HK peptide at 1 h after injection (from 0.43 ± 0.09 to 0.24 ± 0.04 %ID/g, *n* = 4, *P *< 0.01).

The uptake of ^99m^Tc–HYNIC–cHK was similar to ^99m^Tc–HYNIC–HK in BxPC-3 tumors at 0.5 h after injection (0.63 ± 0.18 vs. 0.58 ± 0.09, *n* = 4, *P *> 0.05). However, the uptake of ^99m^Tc–HYNIC–cHK in muscle or bone was much lower, and the tumor-to-muscle (T/M) ratio was significantly higher than that of ^99m^Tc–HHK (2.99 ± 0.87 vs. 1.82 ± 0.27, *n* = 4, *P *< 0.05; Fig. 3B).

### SPECT imaging of BxPC-3 cancer xenografts

**Fig. 4:**
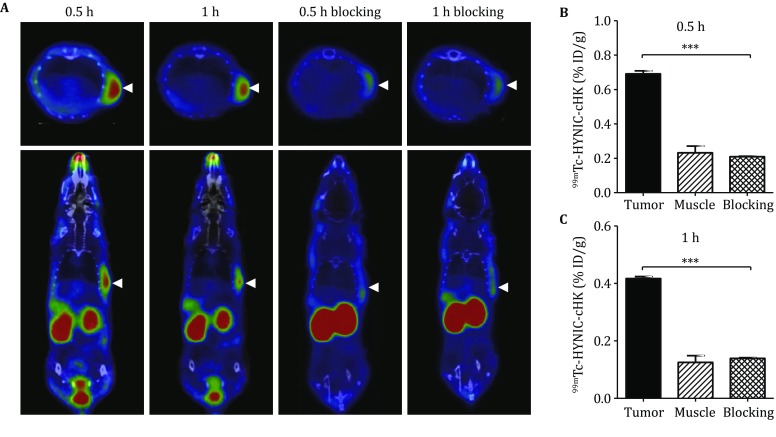
**A** Representative small-animal SPECT/CT images obtained at 0.5 and 1 h after injection of ^99m^Tc–HYNIC–cHK nude mice in BxPC-3 tumor-bearing nude mice without or with blocking dose of cold HK peptide. Arrows indicate the location of tumors. **B**–**C** Quantitation of tumor and muscle uptakes of ^99m^Tc–HYNIC–cHK from SPECT scanning, ^***^P < 0.001

Representative small-animal SPECT/CT images of BxPC-3 tumor-bearing mice at 0.5 h and 1 h after intravenous injection of ^99m^Tc–HYNIC–cHK are shown in Fig. 4A. The radiotracer showed clear tumor imaging with high contrast to the contralateral background. The *in vivo* receptor-binding property of ^99m^Tc–HYNIC–cHK was determined by the blocking study. The tumor uptake of ^99m^Tc–HYNIC–cHK was almost completely inhibited in the HK blocking group (*P *< 0.001; Fig. 4B, C).

### SPECT/CT imaging of bleomycin-induced pulmonary fibrosis

**Fig. 5:**
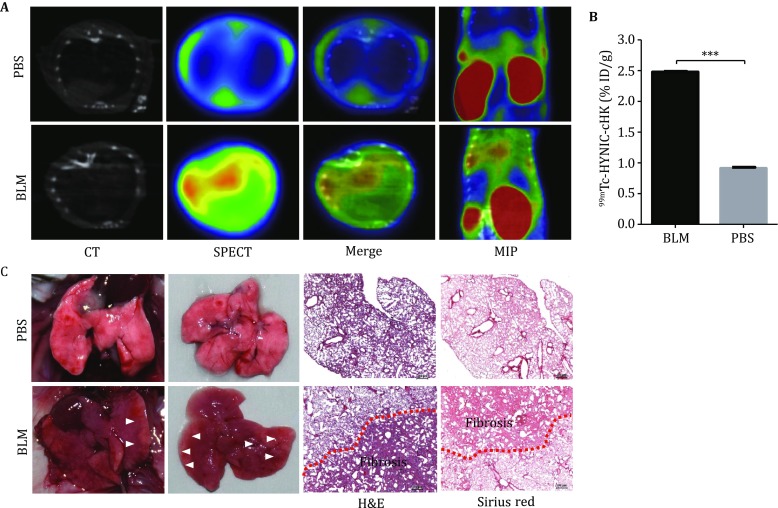
**A** Representative SPECT/CT images of ^99m^Tc–HYNIC–cHK in BLM-treated and PBS-treated C57/BL6 mice at 0.5 h after injection. **B** Quantitation of lung uptakes of ^99m^Tc–HYNIC–cHK in BLM-treated and PBS-treated C57/BL6 mice from the SPECT scanning, ^***^P < 0.001. **C** Gross observation of the lungs, and H&E and Sirius red staining of the lung tissues from BLM-treated and PBS-treated C57/BL6 mice

As shown in Fig. 5A, markedly gray regions were observed by CT imaging in the lung areas of mice in the bleomycin (BLM)-treated mice, suggesting the evident fibrosis formation induced by BLM. Notably, an evident accumulation of ^99m^Tc–HYNIC–cHK in the lungs of the BLM-treated mice was observed. In contrast, no significant uptake of ^99m^Tc–HYNIC–cHK was observed in the phosphate-buffered saline (PBS)-treated mice (Fig. 5A, B). After the SPECT/CT imaging, the mice were sacrificed, and the presence of fibrosis in the edge of pulmonary lobes were verified by anatomic visualization after dissection in the BLM group. The hematoxylin–eosin (H&E) and Sirius red (specific for collagen) staining further confirmed the SPECT/CT findings (Fig. 5C).

## Discussion

Overexpression of integrin αvβ6 has been found in approximately 100% of pancreatic cancers (Liu *et al*. 2014a). Integrin αvβ6-targeted imaging for pancreatic cancer detection and staging would contribute to improve the prospect of curing or controlling pancreatic cancer. We previously synthesized a linear peptide-based SPECT radiotracer (^99m^Tc–HYNIC–HK) and demonstrated its potential for the specific detection of subcutaneous pancreatic tumor xenografts and liver metastases in mouse models (Liu *et al*. 2014b). However, ^99m^Tc–HYNIC–HK had a very poor *in vivo* stability, which may significantly hamper its potential clinical translation. There are several approaches to improve the *in vivo* stability of peptides, including changing some amino acids of the peptide into D-amino acids, cyclizing the peptide to be a cyclic peptide, or engineering the peptide into scaffold-based peptides, such as cysteine knot (Zhu *et al*. 2014). In this study, we synthesized a cyclic peptide based on the HK peptide, and radiolabeled it with ^99m^Tc, the resulting radiotracer ^99m^Tc–HYNIC–cHK was evaluated both *in vitro* and *in vivo*.

Through the *in vitro* solution stability study, ^99m^Tc–HYNIC–cHK was demonstrated to be rather stable in FBS or l-cysteine over 4 h. The *in vivo* metabolic study indicated that the stability in blood was considerably improved after cyclization (Fig. 2). Afterward, the integrin αvβ6-targeting ability of ^99m^Tc–HYNIC–cHK was evaluated through cell-binding assays in integrin αvβ6-positive BxPC-3 cells. Similar to the HK peptide, cHK could also inhibit the binding of ^125^I–HYK on BxPC-3 cells in a dose-dependent manner. However, the binding affinity of cHK to integrin αvβ6 was slightly lower than that of the HK peptide. The decreased affinity may result from the shortened peptide sequence and constrained conformation of the cyclic peptide compared to the linear peptide. ^99m^Tc–HYNIC–cHK retains the integrin αvβ6-targeting capability as evidenced by the significantly inhibited binding by adding an excess of cold cHK or HK peptide (Fig. 1C).

The *in vivo* integrin αvβ6-targeting specificity of ^99m^Tc–HYNIC–cHK was confirmed by the biodistribution and SPECT/CT imaging studies in the BxPC-3 xenograft tumors. ^99m^Tc–HYNIC–cHK exhibited rapid tumor accumulation and showed the maximum tumor-uptake values at 0.5 h after injection (Fig. 3A). Predominant kidney uptake of ^99m^Tc–HYNIC–cHK was also observed, most likely due to the renal clearance of this radiotracer. The absolute tumor uptake of ^99m^Tc–HYNIC–cHK was comparable to that of ^99m^Tc–HHK at 0.5 h (Fig. 3). However, the tumor-to-muscle ratio was significantly higher for ^99m^Tc–HYNIC–cHK compared to that of ^99m^Tc-HHK, resulting in a favorable tumor imaging contrast.

In addition to cancer, *de novo* and increased expression of integrin αvβ6 also occur during fibrogenesis. The increased expression of integrin αvβ6 has been found in fibrotic lung tissue in patients with IPF and was demonstrated to play an important role in the progression of lung fibrotic disease in several different studies (Horan *et al*. 2008; Puthawala *et al*. 2008; Xu *et al*. 2009). To date, the only approach to detect the expression of integrin αvβ6 in the fibrotic lung is immunohistochemical analysis of biopsy samples (Raghu *et al*. 2011). This procedure is clinically impractical for many patients and suffers from sampling bias, resulting in incomplete information. Considering the high short-term mortality following lung biopsy (Utz *et al*. 2001), repeated sampling is unrealistic. Hence, noninvasive molecular imaging of integrin αvβ6 expression would offer a remarkable improvement for immunophenotyping patients with IPF. ^18^F–FDG and ^68^Ga-labeled somatostatin analogs targeting somatostatin receptor as PET tracers have been used for IPF stratification, but neither of these radiotracers targets well-validated pathways implicated in IPF (Ambrosini *et al*. 2010; Win *et al*. 2012). Cai *et al*. recently developed an optical activatable probe for noninvasive diagnosis of IPF by targeting matrix metalloproteinase type 2 (MMP-2), which was also correlated with IPF development. However, MMP-2 could be secreted into the blood stream, the nonspecific fluorescence signal recovery in tissues (*e.g.* liver) other than lung was noticed over time (Cai *et al*. 2013). John *et al*. developed an ^111^In-labeled αvβ6-specific peptide (^111^In–DTPA–A20FMDV2) as a SPECT radiotracer and for the first time used it for noninvasive measurement of integrin αvβ6 expression in lungs of mice with BLM-induced fibrosis (John *et al*. 2013). Their results showed that the lung uptake of ^111^In–DTPA–A20FMDV2 is quantifiable and correlates with the levels of αvβ6 protein, itgb6 messenger RNA, and hydroxyproline in the lungs.

The low uptake of ^99m^Tc–HYNIC–cHK in normal lungs makes it a potential tool for quantitative and global analyses of integrin αvβ6 expression with high sensitivity in imaging the lung disorders. In the murine model of pulmonary fibrosis induced by BLM, a significant accumulating of ^99m^Tc–HYNIC–cHK in lungs of BLM-treated mice was observed, compared with the PBS group (Fig. 5A, B). The H&E and Sirius red staining confirmed the lung fibrotic lesions in the lungs of BLM-treated mice (Fig. 5C). Compared with ^111^In, ^99m^Tc is more suitable for labeling peptide-based probes because that the radioactive half-life of ^99m^Tc matches the metabolic half-life of peptides. Moreover, ^99m^Tc-labeled radiotracer is more widely available and cost effective. The high labeling yield of ^99m^Tc chelator systems also allows the formulation of kits for the rapid preparation of radiotracers for widespread applications.

Although the metabolic stability of ^99m^Tc–HYNIC–cHK after cyclization was significant improved compared to the linear radiotracer ^99m^Tc–HYNIC–HK, the receptor-binding affinity of ^99m^Tc–HYNIC–cHK was slightly decreased. In order to increase the receptor-binding affinity and further improve the *in vivo* pharmacokinetics of ^99m^Tc–HYNIC–cHK, efforts such as polyethylene glycol (PEG)ylation and multimerization may be required to further optimize this radiotracer.

## Conclusion

A cyclic peptide-based radiotracer ^99m^Tc–HYNIC–cHK with improved *in vivo* metabolic stability was prepared and evaluated both *in vitro* and *in vivo*. ^99m^Tc–HYNIC–cHK exhibited specific integrin αvβ6-targeting ability and was demonstrated to specific detection of integrin αvβ6 expression in subcutaneous pancreatic cancer xenografts and pulmonary fibrosis in animal models. Further optimization of ^99m^Tc–HYNIC–cHK may eventually yield a clinical applicable radiotracer for SPECT imaging of integrin αvβ6 expression, disease staging, and monitoring of therapy efficacy.

## Experimental section

### Materials and reagents

All commercially available chemical reagents were used without further purification unless otherwise stated. The peptides Fmoc-cHK, HYK and HK were synthesized by ChinaPeptide Co., Ltd (Shanghai, China). Na^99m^TcO_4_ was obtained from a commercial ^99^Mo/^99m^Tc generator (Beijing Atom High Tech Co., Beijing, China). The reversed-phase high-performance liquid chromatography (HPLC) system was Agilent Technologies 1260 Infinity HPLC (Agilent Technologies, Santa Clara, CA) coupled with the Raytest Gabi radioactivity detector (Raytest, Straubenhardt, Germany). Female BALB/c nude mice (4–5 weeks of age), BALB/c normal mice (4–5 weeks of age), and C57/BL6 mice (7–8 weeks of age) were purchased from Department of Laboratory Animal Science, Peking University Health Science Center (Beijing, China). BLM was purchased from Aladdin (Shanghai, China).

### Synthesis of HYNIC-conjugated cHK peptide

The Fmoc-cHK peptide was conjugated with HYNIC–NHS using a standard procedure. Briefly, a solution of Fmoc-cHK was mixed with HYNIC–NHS at a mole ratio of 1:1.2. The pH was adjusted to 8.5–9.0 using *N*,*N*-Diisopropylethylamine. After stirring for 4 h at room temperature, the Fomc was removed by adding piperidine with a final volume fraction of 20%. The HYNIC–cHK was isolated by semi-preparative HPLC and lyophilized to afford the final product as a white powder (yield: 56%). Analytical HPLC (Retention time = 14.99 min) and mass spectrometry (MALDI-TOF–MS: *m*/*z*, 1380.66 for [MH]^+^ (C_56_H_85_N_17_O_18_S_3_, calculated molecular weight 1380.57) confirmed the identity of the product.

### Preparation of ^99m^Tc–HYNIC–cHK

For ^99m^Tc labeling, a mixture of 20 mg tricine (100 mg/mL in 25 mmol/L succinate buffer, pH 5.0), 740 MBq (20 mCi) Na^99m^TcO_4_, and 30 μl SnCl_2_ (1 μg/μL in 0.1 mol/L HCl) was successively added to 20 μg of HYNIC–cHK with constant stirring at 99 °C for 10 min. Then 100 μl of ethylenediamine-*N*,*N*′-diacetic acid (100 mg/mL) was added to the mixture with contant stirring at 99 °C for 20 min. After allowing it to cool down to room temperature, the mixture was purified with Sep-Pak C18 cartridges (Waters) as preciously described (Jia *et al*. 2006).

### Cell culture and animal models

The BxPC-3 human pancreatic cancer cell line was obtained from American Type Culture Collection. Cells were cultured in RPMI-1640 medium supplemented with 10% FBS at 37 °C in humidified atmosphere containing 5% CO_2_.

All animal experiments were performed in accordance with the guidelines of Peking University Animal Care and Use Committee. To establish the BxPC-3 subcutaneous tumor model, BxPC-3 cells (1 × 10^7^ in 100 μl of PBS) were inoculated subcutaneously into the right front flanks of female BALB/c nude mice. The animals were used for *in vivo* studies when the tumor size reached 200–300 mm^3^ (3–4 weeks after inoculation). For the pulmonary fibrosis mouse model, BLM (1.5 units/kg, 50 μl in PBS) or PBS (50 μl; as a vehicle control) was administered once into the C57/BL6 mice by intratracheal injection. On day 21 (based on pilot studies), the mice with well-established pulmonary fibrosis were used for SPECT/CT imaging.

### Integrin αvβ6 binding specificity

*In vitro* integrin αvβ6 binding affinities of cHK and HK were compared via displacement cell-binding assays (Dong *et al*. 2015) using ^125^I–HYK as the radioligand. ^125^I–HYK was prepared by labeling HYK with Na^125^I using the Iodogen method as previously reported (Liu *et al*. 2010). Experiments were performed on high integrin αvβ6-expressing BxPC-3 cells. The best-fit 50% inhibitory concentration (*IC*_50_) values were calculated by fitting the data with nonlinear regression using Graph-Pad Prism (GraphPad Software, Inc.).

*In vitro* integrin αvβ6 binding specificity of ^99m^Tc–HYNIC–cHK was tested using the integrin αvβ6-positive BxPC-3 cells. Briefly, cells were seeded into 12-well plates and incubated overnight at 37 °C to allow adherence. After brief washing with PBS, tumor cells were incubated with 3.7 kBq ^99m^Tc–HYNIC–cHK with or without an excess dose of cold cHK or HK peptide at 4 °C for 4 h. Tumor cells were then washed with chilled PBS and harvested by trypsinization with 0.05% trypsin. The cell suspensions were collected and measured in a γ-counter (Wallac 1470-002, Perkin-Elmer, Finland). The cell uptake was expressed as the percent added dose (%AD). Experiments were performed twice with triplicate samples.

### Solution and metabolic stability

^99m^Tc–HYNIC–cHK was incubated in FBS or l-cysteine (1.0 mg/mL) for 0, 0.5, 1, 2, and 4 h at 37 °C to test the *in vitro* solution stability. After passing through a 0.22-μm Millipore filter, the samples were analyzed by radio-HPLC.

The metabolic stability of ^99m^Tc–HYNIC–cHK was evaluated in female BALB/c normal mice. Each mouse was administered with the radiotracer at a dose of 1 mCi in 100 μl saline via intravenous injection. At 0.5 h and 1 h postinjection, the blood and urine samples were collected. The samples were centrifuged at 8000 r/min for 15 min. The supernatant was collected, filtered through a 0.22-μm Millipore filter, and then analyzed by radio-HPLC.

### Biodistribution

Biodistribution studies were performed using female BALB/c nude mice bearing BxPC-3 xenografts. Mice received an injection via the tail vein of 370 kBq (10 μCi) of ^99m^Tc–HYNIC–cHK to evaluate the distribution of the radiotracer. The blocking experiments were also performed by coinjection of ^99m^Tc–HYNIC–cHK with a saturating dose of the HK peptide (500 μg per mouse). At 0.5, 1, and 2 h after injection, the animals were sacrificed, and the tumors and the organs/tissues of interest were dissected and wet-weighed, and the radioactivity in the tissue was measured using a γ-counter. The results are presented as percentages of injected dose per gram of tissue (%ID/g). Values are expressed as mean ± SD (*n* = 4 per group).

### Small-animal SPECT/CT imaging

Small-animal SPECT/CT scans of subcutaneous BxPC-3 tumor and pulmonary fibrosis mouse models were performed using a SPECT/CT system (NanoScan; Mediso, Budapest, Hungary). Each BxPC-3-bearing mouse was injected via tail vein with 37 MBq (1 mCi) of ^99m^Tc–HYNIC–cHK. At 0.5 h and 1 h after injection, the mice were anesthetized by inhalation of 2% isoflurane and imaged using the Nano-SPECT/CT camera, The SPECT and CT fusion images were obtained using the automatic fusion software (InterView Fusion; Mediso Medical Imaging Systems, Budapest, Hungary).

Each BLM- or PBS-treated mouse was administered with 37 MBq of ^99m^Tc–HYNIC–cHK via tail vein. After SPECT/CT imaging, the BLM- and PBS-treated mice were euthanized. Lungs were excised and fixed in 5% buffered formalin, embedded in paraffin, and cut into sections for staining with H&E or Sirius red as previously described (Yu *et al*. 2016).

### Statistical analysis

Quantitative data were expressed as mean ± SD. Results were compared using the Student *t* test. *P* values of less than 0.05 were considered statistically significant.
